# Morphological and molecular identification of amphistomes occurring in cattle in Zimbabwe

**DOI:** 10.1007/s00436-026-08682-6

**Published:** 2026-05-07

**Authors:** Ignore Nyagura, Mokgadi P. Malatji, Madeline S. Sibula, Philile I. Ngcamphalala, Samson Mukaratirwa

**Affiliations:** 1 School of Life Sciences, College of Agriculture, Engineering and Science, University of KwaZulu-Natal, Westville Campus, Durban, 4001 South Africa; 2 National University of Science and Technology, P. Bag AC939, Ascot, Bulawayo, Zimbabwe; 3 One Health Centre for Zoonoses and Tropical Veterinary Medicine, Ross University School of Veterinary Medicine, Basseterre, West Indies St Kitts And Nevis

**Keywords:** Cattle, Geographical distribution, Amphistomes, Morphology, ITS-2, *Calicophoron*, *Carmyerius*, *Cotylophoron*, *Paramphistomum*, Zimbabwe

## Abstract

This study characterized and identified amphistomes collected from cattle slaughtered at selected abattoirs in Zimbabwe, using both morphological and molecular techniques. Amphistomes were collected from 158 cattle originating from 14 localities, slaughtered in abattoirs in Harare and Bulawayo. One-hundred and twenty-one amphistome specimens were selected and sectioned into two portions, one piece for morphological characterization based on histological sectioning using haematoxylin and eosin staining, and the other piece for molecular identification based on the ITS-2 gene. Morphological characters classified the amphistomes into ten species as follows; *Calicophoron (C.) calicophorum*,* C. microbothrium*, *C. clavula*, *C. raja*, *C. phillerouxi*, *C. sukari*, *Paramphistomum (P.) gracile*, *P. hibarniae*,* Carmyerius (Ca.) multivitellarius*, *Cotylophoron (Co.) cotylophorum*, and unidentified *Paramphistomum* sp. *Calicophoron* was the most common genus, and *C. microbothrium* was the most common and widely distributed species recorded across the 14 localities. The ITS-2 gene was able to confirm the identification of *C. microbothrium*, *C. clavula*, *C. raja*,* C. calicophorum* and *C. phillerouxi*. There were no comparative ITS-2 sequences for *C. sukari*, *P. gracile* and *P. hibarniae* on the GenBank. Hence, specimens identified morphologically as *C. sukari *showed a 100% homology to multiple *Calicophoron* species, but grouped with *C. raja.* Furthermore, *Ca. multivitellarius* specimens confirmed the genus identity by forming sister clade with other *Carmyerius* spp. There were no species-level resolution for *Paramphistomum* sp., *P. gracile* and *P. hibarniae*, as these isolates formed a clade with *Paramphistomum* sp. (OQ459353.1). The study resulted in new geographical records for *P. gracile*, *P. hibarniae* and *Ca. multivitellarius* in Zimbabwe, hence adding new knowledge to the geographical distribution of these species within Africa.

**Supplementary information:**

The online version contains supplementary material available at 10.1007/s00436-026-08682-6.

## Introduction

Amphistomes are parasitic trematodes belonging to the family Paramphistomidae, primarily known to inhabit the rumen and other digestive organs of domestic and wild ruminants (Lotfy et al. 2010). They are of veterinary interest due to their significant effects on the health of domestic and wild ruminants, especially growing animals where they cause amphistomosis (Samn 2017; Hajipour et al. 2021). Amphistome species from the genus *Paramphistomum* (Fischoeder 1901), *Calicophoron* (Näsmark 1937), *Cotylophoron* (Stiles and Goldberger 1910) and *Balanorchis* (Fischoeder 1901) (Pfukenyi and Mukaratirwa 2018; Huson et al. 2017; Tandon et al. 2019) have been reported in ruminants. According to Mas-Coma et al. (2006), some amphistome species belonging to the genera *Watsonius* (Stiles and Goldberger 1910), *Gastrodiscoides* (Leiper 1913), and *Fischoederius* (Stiles and Goldberger ) have been reported to be zoonotic, occasionally infecting humans.

Historically, amphistomes taxonomy was based on morphological features to distinguish between various genus and species, which then provided valuable insights into species classification and biodiversity (Cháves-González et al. 2022; Guo et al. 2022). Foundational studies conducted by Eduardo (1984, 1985), and Sey (1983), have made significant contributions to morphological taxonomy by providing detailed descriptions and diagnostic criteria for many amphistome taxa. These studies were based on variable morphological features of adult flukes, which when used alone have shown not to be reliable, resulting in misidentification, particularly for cryptic species found in some of the genera (Horak 1971; Jones 2005; Mage et al. 2002; Rinaldi et al. 2005).

In Africa, taxonomic studies of amphistomes in both livestock and wildlife has been conducted based on morphological features visualised through histology (Dinnik and Dinnik 1954; Dinnik 1961, 1965; Wright et al. 1979; Southgate et al. 1985). Despite the widespread use of the histological technique, identifying amphistomes to species level remains controversial (Pfukenyi and Mukaratirwa 2018). Mitchell et al. (2021) also pointed out that morphological plasticity caused an array of misinterpretations in amphistome identification and furthermore, the thick and robust bodies of amphistomes which obscure internal organs also contributes further to the difficulties in their identification (Lotfy et al. 2010). Moreover, identifying species responsible for severe cases of amphistomosis is also even more difficult as the flukes responsible are often sexually immature, and lack the distinctive features typically used for morphological classification (Horak 1971).

The morphological limitations have emphasized the importance of molecular approaches, which have lately emerged as significant tools for taxonomic classification (Tookhy et al. 2022). These techniques have been used for identification of amphistomes to species level using several genes such as the nuclear internal transcriber spacer (rDNA ITS-2), and the mitochondrial cytochrome c oxidase I (*cox1*). Of the two genes, the ITS-2 has been deemed to be more precise in identifying species and studying their phylogenetic relationships (Itagaki et al. 2003; Martinez-Ibeas et al. 2013; Samn 2017; Laidemitt et al. 2017; Rafiq et al. 2020). According to Nolan and Cribb (2005), these methods are thought to have the abilities to resolve taxonomic ambiguities and distinguish cryptic species that are morphologically similar but genetically distinct. Although a few studies have been conducted on the higher-level classes among paramphistomoid in Africa (Dube et al. 2015; Laidemitt et al. 2017; Paguem et al. 2023), Alves et al. (2020) highlighted that the scarcity of molecular data, particularly from major genera and families, impedes progress in understanding the interrelationships within this superfamily. Benovics et al. (2022) also indicated that genetic databases may not adequately represent the global diversity of amphistomes, making it difficult to link novel sequences with existing ones, resulting in ambiguous or inconclusive identification. Furthermore, if a novel parasite sequence does not closely match any existing sequences, it may be misinterpreted or overlooked as a separate species (Goswami et al. 2009). As a result, focussing entirely on molecular analysis increases the danger of misclassifying parasites that have not been well characterised at the morphological level (Alves et al. 2020).

Thus, a more effective strategy involves combining molecular and morphological data to improve our understanding of amphistome classification and diversity (Sibula et al. 2024). In this context, the study used the morphological and molecular techniques with the aim of identifying amphistome species collected from cattle in Zimbabwe. The ITS-2 gene was selected in this study due to its vast use and recommendation for identifying amphistomes and studying their phylogenetic relationships, and the present of more GenBank representation of African amphistome species.

## Materials and methods

### Study area and sample collection

Amphistomes were collected from the rumen and reticulum of 158 adult cattle slaughtered at abattoirs in Harare and Bulawayo, Zimbabwe, between 2022 and 2023. The cattle originated from diverse ecological regions, each with distinct climatic conditions, i.e., region 1 which included Manicaland and parts of Mashonaland provinces is characterized by high rainfall, temperate climates, and fertile soils conducive to agriculture and livestock farming. Bulawayo, Matabeleland North, Matabeleland South, and Masvingo provinces fall within Natural Region IV, which is characterized by low rainfall and semi-arid conditions. The Midlands province is classified under Natural Region III and has low to moderate rainfall. Cattle were randomly sampled during routine meat inspection from abattoirs located in the following provinces: Bulawayo (10 cattle), Matabeleland North (Umguza = 6, Nkayi = 4, Nyamandlovu = 15, Lupane = 10), Matabeleland South (Gwanda = 9), Mashonaland East (Nyamapanda = 12, Mutoko = 17, Uzumba = 16), Manicaland (Murambinda = 7, Mutasa = 10, Nyanga = 8, and Headlands = 14), Masvingo (Gutu = 8), and Midlands (Mvuma = 12). Cattle from Manicaland, Mashonaland, Midlands, and Masvingo were slaughtered at Koala Park Butchery and Abattoir in Harare, while those from Bulawayo, Matabeleland North, and Matabeleland South were slaughtered at CSC and Grills abattoirs in Bulawayo. The intensity of amphistomes in hosts from which collection was done ranged from 10 to 300 specimens per cattle. Depending on the abundance of amphistomes in each animal selected, 5 to 10 specimens were randomly collected per cattle, washed with normal saline and stored in 70% ethanol until further analysis.

### Morphological characterization

A total of 121 amphistome specimens collected from the following provinces in Zimbabwe; Bulawayo (*n* = 4), Matabeleland North (*n* = 18), Matabeleland South (*n* = 2), Midlands (*n* = 7), Mashonaland East (*n* = 46), Manicaland (40), and Masvingo (*n* = 4), were selected for histological sectioning based on their size, shape, and colour. Each specimen was sectioned into two segments; the smaller segment was stored for DNA extraction, and the larger segments underwent dehydration process through a series of ethanol at increasing concentrations (70–100%), and subsequently cleared in xylene before being embedded in paraffin wax. The embedded specimens were cut into thin (5 μm) pieces with hand microtome (R Jung AG Heidelberg, Germany) and placed on glass slides for additional examination. Haematoxylin and eosin (H&E) stain was used for both identification of anatomical features and general histological characterization. The sections were examined under a light microscope (Carl Zeiss, West Germany), and each segment was photographed using a 50 megapixel front camera. The acetabulum, pharynx, and occasionally the genital atrium and ventral pouch were used to characterize the various species, as described by Nasmark (1937), Eduardo (1982, 1983, 1984), Sey (1983, 2019).

### Molecular analysis

DNA was extracted from 28 representative specimens (each matching specimen identified morphologically) using the Qiagen DNeasy Blood and Tissue Kit (Qiagen, Germany) following the manufacturer’s protocol. Using the nuclear ribosomal rDNA ITS-2 primers GA1 (5’-AGAACATCGACATCTTGAAC-3’) and BD2 (5’ – TATGCTTAAATTCAGCGGGT-3’) (Laidemitt et al. 2017), PCR amplification was performed in a 25 µL reaction volume, containing 12.5 µL of 2x Taq polymerase buffer (NEB, England), 1 µL each of the primers (10 µM), 7.5 µL of nuclease-free water, and 3 µL (~ 50 µg) of template DNA. The PCR cycling conditions were set at 95 °C initial denaturation for 3 min, followed by 30 cycles of denaturation at 95 °C for 30 s, annealing at 52 °C for the primers for 45 s, and extension at 72 °C for 1 min, with a final extension at 72 °C for 15 min. The resulting amplicons were run on a 2% agarose gel, stained with SYBR Safe DNA Gel Stain (Applied Biosystems, USA), and successful samples were visualized by a band at approximately 380 bp. Amplicons were then sent to Inqaba Biotechnical Industries (Pretoria, South Africa) for sanger sequencing.

### Phylogenetic analysis

The forward and reverse sequences were assembled and consensus sequences created on BioEdit program (Sequence Alignment Editor version 7.2) (Hall 1999). Multiple alignment with homologous sequences obtained from the GenBank database was performed using Clustal W option on BioEdit (Thompson et al. 1997) and then trimmed to a common length on 380 nucleotides. jModeltest 2.355 (Posada 2008) selected the Hasegawa-Kishino-Yano (HKY + G) model as the best model fit for nucleotide substitution for the dataset. PAUP* 4.07 (Swofford 2002) was used to generate the maximum likelihood phylogram and the nodal support was estimated using 1,000 pseudo-replicates. Bayesian inference tree was executed on MrBayes 3.1.2 (Huelsenbeck and Ronquist 2001), following the four Markov chain, which ran for 5 million generations, until the standard deviation of the split frequencies was less than 0.01 and discarded the first 5000, 000 trees as burn-in.

## Results

### Morphological characterization

Using morphological features, 10 species were identified across four genera as *Calicophoron* (*C.*) *calicophorum* (*n* = 2), *C. microbothrium* (*n* = 57), *C. clavula* (*n* = 22), *C. raja* (*n* = 17), *C. phillerouxi* (*n* = 1), C. sukari (*n* = 2), *Paramphistomum* (*P.*) *gracile* (*n* = 4), *P. hibarniae* (*n* = 6), *Paramphistomum* species (*n* = 7), *Carmyerius* (*Ca.*) *multivellarius* (*n* = 2) and *Cotylophoron* (*Co.*) *cotylophorum* (*n* = 1). The salient features used for identification and distinguishing between species are highlighted in Table 1 and Supplementary Fig. S1, and included the acetabulum type, pharynx type, genital atrium type (if present) and positioning of the testes in the body of the median sagittal sections.

**Table 1:** Identification of amphistomes collected from cattle abattoir in Zimbabwe based on morphological characters

Provisional identification	No. of specimens	Acetabulum type	Pharynx type	Genital sucker present and type	Size and position of testes	Ventral pouch	Confirmed by rDNA ITS-2 gene	Reference
*Calicophoron calicophorum*	2	*Calicophoron*	*Calicophoron*	*Calicophoron*	Testes fairly large, in tandem and middle in the body	No	No	Eduardo 1983; Sey 2019
*Calicophoron clavula*	22	*Paramphistomum*	*Calicophoron*	*Calicophoron*	Testes large, deeply lobed and in posterior two thirds of the body	No	Yes	Eduardo 1983; Sey 2019
*Calicophoron phillerouxi*	1	*Paramphistomum*	*Calicophoron*	Microbothrium	Testes very large, and in posterior two thirds of the body	No	Yes	Eduardo 1983; Sey 2019
*Calicophoron microbothrium*	57	*Paramphistomum*	*Calicophoron*	Microbothrium	Testes fairly large and in posterior half of the body.	No	Yes	Eduardo 1983; Sey 2019
*Calicophoron raja*	17	Pisum	*Calicophoron*	Raja	Testes fairly large and in posterior half of the body.	No	Yes	Eduardo 1983; Sey 2019
*Calicophoron sukari*	2	*Calicophoron*	*Calicophoron*	Microbothrium	Testes fairly large, in posterior two thirds of the body	No	No	Eduardo 1983; Sey 2019
*Cotylophoron cotylophorum*	1	*Cotylophoron*	*Calicophoron*	No	Large and juxtapositioned in middle of the body	No	No	Eduardo 1985; Sey 2019
*Paramphistomum gracile*	4	*Paramphistomum*	*Calicophoron*	Gracile type	Testes fairly small and in posterior half of the body.	No	No	Eduardo, 1982; Sey 2019
*Paramphistomum* sp.	7	*Paramphistomum*	*Calicophoron*	Unknown	Testes fairly small and in posterior two thirds of the body	No	No	Eduardo, 1982; Sey 2019
*Paramphistomum hibarnae*	6	*Paramphistomum*	Liorchis	No	Testes very small and in posterior two thirds of the body.	No	No	Eduardo 1982; Sey 2019
*Carmyerius multivitellarius*	2	*Gastrothylax*	Paramphistomum	No	Testes on either side of the pouch.	Yes	No	Sey 2019

All five *Calicophoron* species identified had *Calicophoron*-type pharynx, however, *C. calicophorum* had an acetabulum, and genital atrium of a *Calicophoron* type and the testes were lying side by side so that through-out the sagittal sections one testes was seen. *Calicophoron microbothrium*,* C. clavula* and *C. phillerouxi* had an acetabulum that is of the paramphistomum type. However, *C. microbothrium* and *C. phillerouxi* displayed a microbothrium-type genital atrium, while *C. clavula* genital atrium was of the clavula type. The testes were deeply lobbed in all three species but were directly tandem on posterior half of the body in *C. microbothrium*, obliquely in tandem on posterior two thirds of the body in *C. clavula*, and lastly obliquely tandem and seemed relatively larger compared to other *Calicophoron* spp. in *C. phillerouxi*. *Calicophoron raja* was identified by a pisum-type acetabulum, a terminal genitalium which is a raja-type with some specimens having a genital pillar. The testes were also lobbed and were obliquely tandem being on the posterior half of the body.

*Cotylophoron cotylophorum* had a *Cotylophoron*-type acetabulum and pharynx which were terminal and of the *Calicophoron* type. This species lacked the genital sucker, and the testes were large and juxtapositioned in middle of the body. The pouched species *Ca. multivitellarius* had the acetabulum typical of *Gastrothylax* but the *Paramphistomum*-type pharynx. The specimens also lacked the genital sucker. Their testes were observed on either side of the pouch, and the ventral pouch was characterised by large tissue invagination. *Paramphistomum gracile* and *P. hibarniae* had a *Paramphistomum* type acetabulum, nearly straight with small deeply lobbed testes that were distance from each other. However, *P. gracile* was slightly longer than *P. hibarniae* and the position of the genital atrium in *P. gracile* was further from the oesophageal bifurcation. *Paramphistomum gracile* had a *Calicophoron* type pharynx and gracile type atrium, but *P. hibarniae* had a liorchis-type pharynx and a leydeni-type genitalium. Additionally, there were 7 specimens that were identified to genus level as *Paramphistomum* sp. These specimens had distinctly small testes that were inconspicuous at first but became visible in the sagittal median section. The genital atrium showed peculiar multiple projections which had well developed sphincter muscles on the inside, with a swollen bulge on the outside with a less developed sphincter muscle.

### Molecular confirmation

**Fig. 1:**
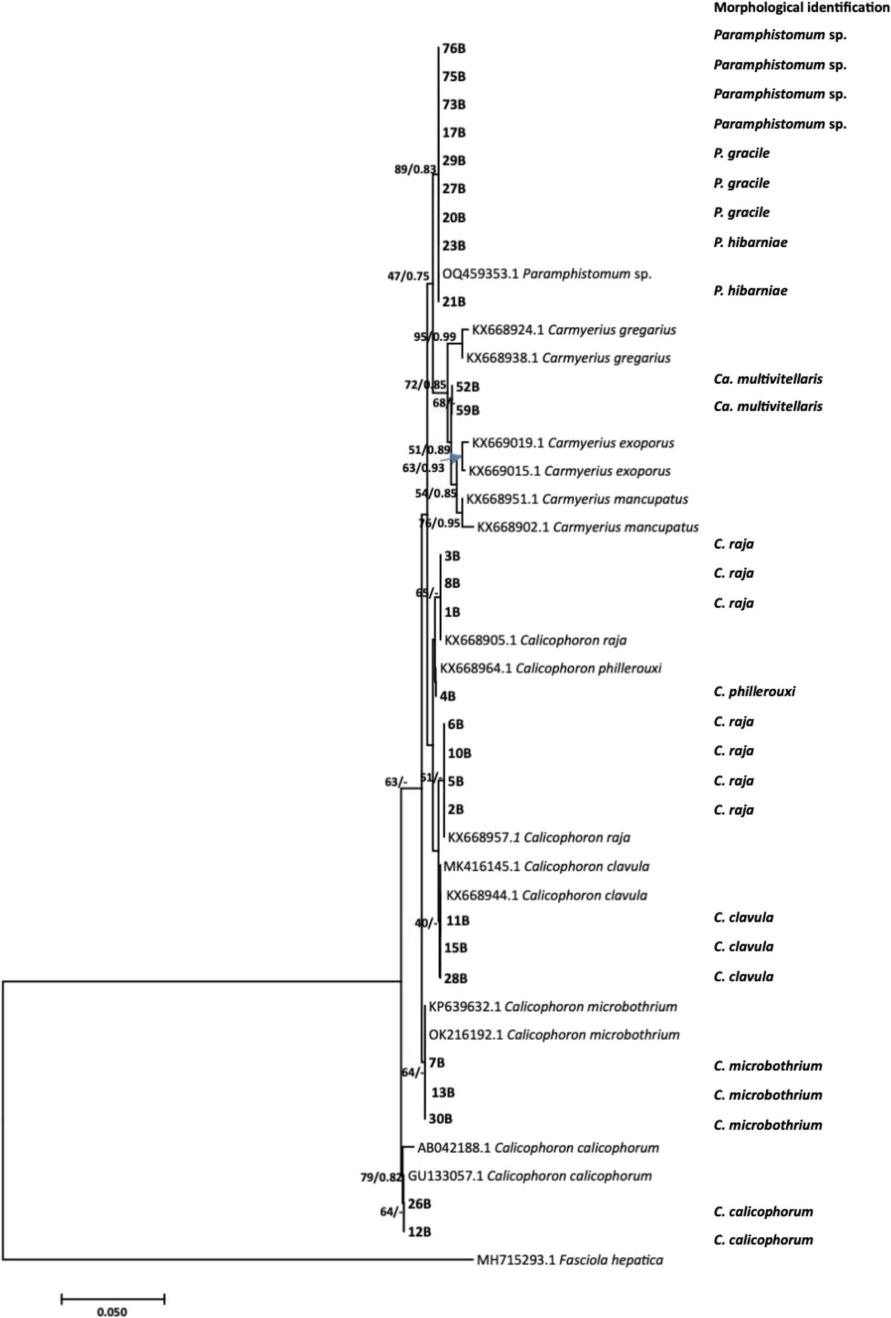
Neighbor-joining tree based ITS2 gene illustrating the relationship amphistomes obtained from different localities and abattoirs in Zimbabwe, and the closest matches from the NCBI GenBank. The nodal support values indicated in the order: neighbour-joining and maximum likelihood

Phylogenetic analysis weakly supported the separation of *Calicophoron* genus and other genera such as the *Carmyerius* and *Paramphistomum* (Fig. 1). The analysis confirmed the morphological identification of *C. microbothrium*, *C. raja*, *C. clavula*, *C. phillerouxi* and *C. calicophorum*, which clearly formed species-specific clades, although weakly supported, with an exception to *C. calicophorum*. There were no *C. sukari* sequences available on GenBank based on the ITS-2, and the isolate (10B) morphologically identified as *C. sukari* showed a 100% similarity with several *Calicophoron* species (*C. microbothrium*,* C. phillerouxi* and *C. raja*), but further formed a clade with *C. raja* (KX668957.1) isolates from Kenya.

Although there were no reference sequences for *P. hibarniae*,* P. gracile* and *Ca. multivitellarius* on the GenBank to compare with, the phylogeny clearly distinguished these species from the *Calicophoron* spp. Maximum likelihood and Bayesian inference moderately supported the grouping of isolates morphologically identified as *P. hibarniae*,* P. gracile* (*n* = 3), and one *Paramphistomum* sp. which was not resolved to species level morphologically (17B) with *Paraphistomum* sp. (OQ459353.1). The two pouched amphistomes, *Ca. multivitellarius* specimens grouped with *Ca. gregarious*,* Ca. exoporus*, and *Ca. mancupatus*, as the former species had no reference sequences on the GenBank. No sequences were generated for *Co. cotylophorum*.

### Distribution of amphistome species

The distribution of amphistome species across the sampled provinces as shown in Table 2 revealed that the *Calicophoron* genus was more common and contributed over 80% of the specimens morphologically identified. *Calicophoron microbothrium* was the most widespread occurring in all sampled provinces, followed by *C. raja* which was present in 71.43% of the sampled provinces. *Calicophoron calicophorum*,* C. phillerouxi*,* C. sukari*,* Co. cotylophorum*,* P. gracile* and *Ca multivitellarius* were the least occurring species, each found in one province. Manicaland and Mashonaland East provinces had the highest amphistome species diversity, and these were the only provinces which documented the *Paramphistomum* species and the pouched *Ca. multivitellarius.* The Midlands province had one species reported, making it the province with the least species diversity.

**Table 2:** Distribution of amphistome species from cattle from different provinces in Zimbabwe identified based on histo-morphology

Province	Locality	N	*C.ca*	*C. cl *	*C. ph*	*C. mi*	*C. ra*	*C. su*	*Cot. co*	*P. gr*	*P. hi*	*P. sp.*	*Ca mu *
Bulawayo Province	Bulawayo	4			1	2	1						
Matebele-land North	UMguza	2				1	1						
	Nkayi	3		1		1	1						
	Nyamandlovu	4	2			1			1				
	Lupane	9		2		5	2						
Matebele-land South	Gwanda	2				1	1						
Midlands Province	Mvuma	7				7							
Mashona-land East	Mutoko	9		3		3	2				1		
	Uzumba	14		5		7	2						
	Nyamapanda	23		5		10	3				3	2	
Manicaland Province	Murambinda	7		2		4	1						
	Mutasa	8				3	2				2	1	
	Nyanga	21		4		5	1	2		4		3	2
	Headlands	4				3						1	
Masvingo Province	Gutu	4				4							
	Total specimens	121	2	22	1	57	17	2	1	4	6	7	2

*N* total number of specimens, *C. ca Calicophoron calicophorum*, *C. cl Calicophoron clavula*, *C.ph Calicophoron phillerouxi*, *C. mi Calicophoron microbothrium*, *C. ra Calicophoron raja*, *C. su Calicophoron sukari*, *Cot. Co* *Cotylophoron cotylophorum*, *P. gr Paramphistomum gracile*, *P. hi Paramphistomum hibarne*, *P. sp Paramphistomum* sp.,*C. mu* *C. multivitellarius*

## Discussion

This study provides important insights into the identification of amphistome species of cattle from Zimbabwe using morphological characters supported by molecular confirmation. To date, 16 amphistome species have been documented in both domestic and wild ruminants in Zimbabwe (Dube et al. 2002, 2004, 2010; Dube and Tizauone 2014; Dube et al. 2015; Pfukenyi and Mukaratirwa 2018), including the new reports of *Gastrothylax crumenifer*, *Orthocoelium dicranocoelium* and *Leiperocotyle gretillati* in wild ruminants (Sibula et al. 2025). The present study morphologically identified ten amphistome occurring in cattle to species level, i.e., *C. microbothrium*, *C. clavula*, *C. phillerouxi*, *C. raja*, *C. calicophorum*, *C. sukari*, *Co. cotylophorum*, *P. gracile*, *P. hibarnae* and *Ca. multivitellarius.* As expected, the *Calicophoron* genus was the most common and diverse of the genera reported occurring in cattle and accounted for more than 80% to the total number of amphistome specimens collected and identified. Similar observation was made in wild ruminants in Zimbabwe, whereby the genus *Calicophoron* genus accounted for more than 80% of the analysed amphistome species, and *C. microbothrium* was identified in all sampled provinces (Sibula et al. 2025). Furthermore, Pfukenyi and Mukaratirwa (2018) previously highlighted that *Calicophoron* species are generally widely distributed in several countries as compared to other genera. Additionally, the authors also indicated that *C. microbothrium* was the most geographically distributed species (Pfukenyi and Mukaratirwa 2018), as observed in the current study. The wide distribution of this species underscores its ecological adaptability to the snail intermediate hosts and highlights its significant role in the epidemiology of amphistomosis in Zimbabwe, as it remains the most encountered species in African livestock (Pfukenyi et al. 2005; Pfukenyi and Mukaratirwa 2018).

Manicaland and Mashonaland East provinces recorded the highest species diversity compared to other regions, accounting for more than 60% of the recorded species with exception to *Co. cotylophorum, C. sukari* and *C. calicophoron* which were recorded in Nyanga, and *C. phillerouxi* recorded in Bulawayo. These provinces have favourable climatic conditions, including moderate to high rainfall, which support a variety of freshwater habitats essential for the breeding of snail intermediate host(s) which are important in the transmission of amphistomes (Pfukenyi et al. 2005; Nyagura et al. 2024). Conversely, the low species diversity displayed in Matebeleland South, Midlands, and Masvingo provinces may likely result from their hotter, drier climates and limited freshwater habitats. These harsh environmental conditions restrict the availability of suitable habitats for intermediate hosts and impede the completion of life cycle, resulting in reduced species richness (Pfukenyi et al. 2005).

Although molecular analysis confirmed the identification of *Calicophoron* species and clearly distinguished these species with *Carmyerius* and *Paramphistomum* species, the sub-clustering of these *Calicophoron* species was weakly supported. Furthermore, despite *C. sukari* previously recorded in Zimbabwe (Pfukenyi and Mukaratirwa 2018), the lack of reference sequences on the GenBank resulted in this not confirmed molecularly, thus highlighting a significant gap in molecular resources for African amphistome species.

This study also documented for the first time *P. gracile* and *P. hiberniae* in cattle in Zimbabwe and Africa. These species have been previously documented in Europe and Asia (Willmott and Pester 1955; Panyarachun et al. 2013). Livestock importation and/or the movement of intermediate hosts across regions may have attributed to the introduction of these species in Zimbabwe (Carolus et al. 2019). While molecular analyses showed that *P. hiberniae*, *P. gracile* and unidentified *Paramphistomum* spp. are closely related and clustered with *Paramphistomum* sp. from Cambodia, the morphological characteristics of *P. hibarniae* and *P. gracile* were however distinct from each other and the unidentified *Paramphistomum* spp. (Eduardo 1982). *Carmyerius multivitellarius*, a pouched species previously reported in Africa (Sey 2019), was also documented for the first time in Zimbabwe. Although sequencing could not identify this specimen to species level, the phylogenetic tree confirmed that the genus level identification of *Ca. miltivitellaius isolates*, by forming a sister clade with other *Carmyerius* species. Thus, correlating with the morphology of these specimens, which was characterised by the presence of a ventral pouch, and other features typical of *Ca. multivitellarius* (Sey 2019).

These results further highlight the discrepancies between morphological data and ITS-2 sequences for some African amphistome species. This was concerning as the ITS-2 primers have been widely used in amphistome research across continents and has variety of representative sequences accessible in GenBank (Goswami et al. 2009; Lofty et al. 2010; Laidemitt et al. 2017), making it an appropriate marker for initial species identification. Furthermore, while the COX1 and other genes such as 28S have been recently used to identify amphistomes, there are less reference sequences on GenBank for comparison albeit their potential excellent inter-species resolution. Therefore, it is critical that reference sequences be curated for all African paramphistomes using various genes, incorporating morphological analysis to confirm species identification and improve taxonomic resolution.

## Conclusions

This study provides valuable insights into the diversity and complexity of amphistome species in Zimbabwe and probably the rest of the African continent, particularly within the genus *Calicophoron*. Our findings emphasize the importance of complementing morphological technique with molecular techniques for accurate identification of amphistomes to species level. The putative identification of *P. gracile*, *P. hibarniae*, and *Ca. multivitellarius* as new geographical records in Zimbabwe further underscores the need for comprehensive surveys to elucidate the taxonomy, distribution, and epidemiology of these parasites. Results from this study contribute new knowledge on the diversity of amphistome species in cattle in Zimbabwe and lay a solid foundation for future research aimed at mapping the spatial distribution of amphistome species of cattle and other ruminant hosts in Zimbabwe in order to design and integrated control program with other snail-borne trematodes of livestock such as fasciolosis and schistosomiasis following a One Health approach.

We acknowledge limitations of our study which include the fact that sampling was conducted as a once-off event and did not cover multiple seasons. Furthermore, the number of cattle sampled from some provinces was relatively small and therefore does not allow inference of true prevalence or representativeness at the local or provincial level. We further clarify that the primary objective of this study was not to estimate prevalence, but rather to investigate the identity of amphistomes to species level based on application of morphological and molecular techniques. We recommend that future studies aiming at prevalence estimation should employ longitudinal and statistically powered sampling designs. Further studies should also assess the epidemiological patterns and disease burden of amphistomes, considering factors such as seasonality, host sex, and age, which were not considered in this study. We further recommend that for reliable and accurate identification of this group of parasites, molecular techniques should be coupled with morphology to avoid spurious identifications. For morphological characterisation, it is recommended to use all diagnostic features including the genital atrium, as these features are critical in distinguishing closely related species.

## Supplementary information

Below is the link to the electronic supplementary material.

Supplementary File 1 (DOCX 2.67 MB)

## Data Availability

The studied specimens were deposited at the University of Kwazulu-Natal Parasitology Laboratory. The sequences generated in this study have been deposited in the NCBI, under the following accession numbers: PV072861- PV072887 (ITS-2 gene).
