# Supplementary material for: Morphological and molecular identification of amphistomes occurring in cattle in Zimbabwe
Source: Parasitol Res. 2026 May 7;125(1):76. doi: 10.1007/s00436-026-08682-6 (PMC13319246; doi:10.1007/s00436-026-08682-6)
Supplement: Supplementary file 1 — (DOCX 2.67 MB) [file 436_2026_8682_MOESM1_ESM.docx]

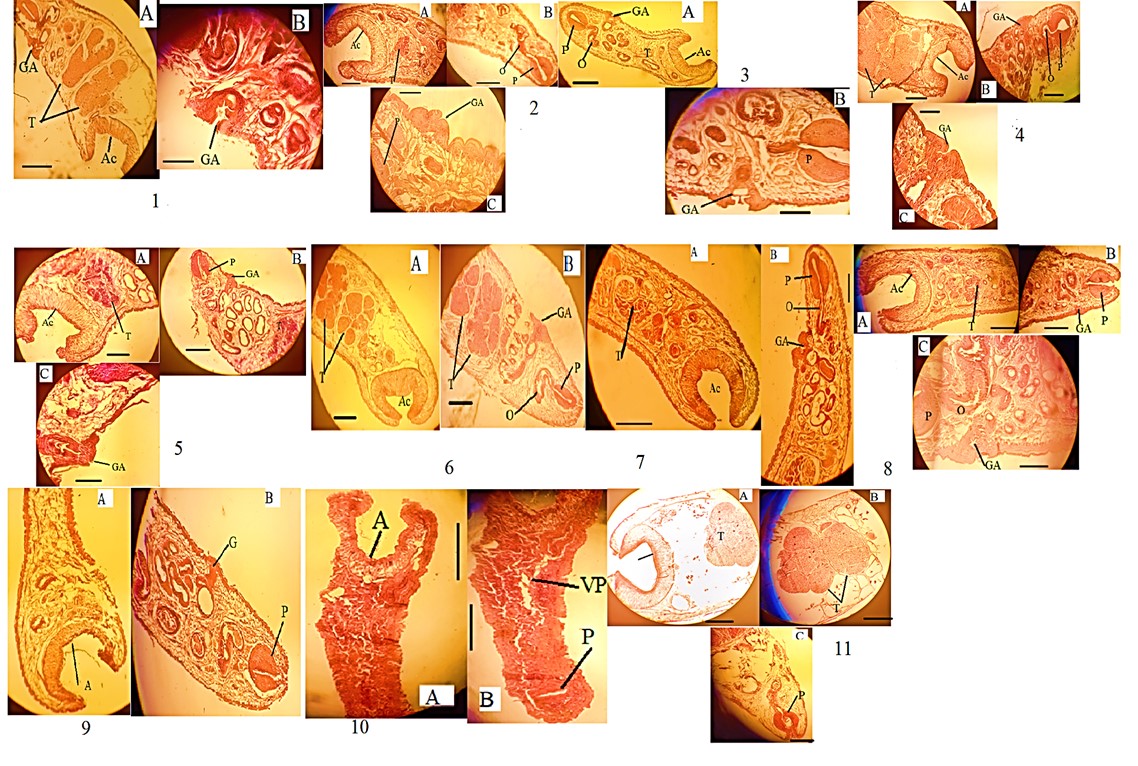


**Supplementary Fig. 1** Representative median sections of eight amphistome species (1-8) identified in wild ruminants in Matebeleland region of Zimbabwe. The amphistome species are: 1. *Calicophoron* (C.) *clavula;* 2. *C. raja;* 3. *C. microbothrium;* 4. *C. phillerouxi;* 5. *C. calicophorum;* 6. *C. sukari;* 7. *Paramphistomum (P.) sp.;* 8*. P. hibarnae;* 9. *P. gracile* 10. *Carmyerius multivitellarius;* 11. *Cotylophoron cotylophorum.* Plate A has the acetabulum (A) and testes (T), Plate B has the pharynx (P), and Plate C has the genital atrium (G). Please note that amphistome number 10 (*Carmyerius multivitellarius*) has a ventral pouch (VP). Scale bars on plates A and B are 1mm whilst that of C is 0.2mm
